# Supplementary material for: A self-referenced in-situ arrival time monitor for X-ray free-electron lasers
Source: Sci Rep. 2021 Feb 11;11:3562. doi: 10.1038/s41598-021-82597-3 (PMC7878505; doi:10.1038/s41598-021-82597-3)
Supplement: Supplementary file 1 — Supplementary Information [file 41598_2021_82597_MOESM1_ESM.pdf]

A self-referenced in-situ arrival time monitor for X-ray free-electron lasers:

Supplementary Information

Michael Diez[1,2,*], Andreas Galler[1,2], Sebastian Schulz[1,2,10,*], Christina Boemer[1,10], Ryan N. Coffee[3], Nick Hartmann[3,4], Rupert Heider[5], Martin S. Wagner[5], Wolfram Helml[5,6,11], Tetsuo Katayama[7,8], Tokushi Sato[9,12], Takahiro Sato[3], Makina Yabashi[7,8], Christian Bressler[1,2,*]

[1] *European XFEL GmbH, Holzkoppel 4, 22869 Schenefeld, Germany*

[2] *The Hamburg Centre for Ultrafast Imaging, Luruper Chaussee 149, 22761 Hamburg, Germany*

[3] *SLAC National Accelerator Laboratory, 2575 Sand Hill Rd., Menlo Park, CA 94025, USA*

[4] *Coherent, Inc., 5100 Patrick Henry Dr., Santa Clara, CA 95054, USA*

[5] *Physik-Department E11, Technical University of Munich, James-Franck-Str. 1, 85748 Garching, Germany*

[6] *Faculty of Physics, Ludwig-Maximilians-Universität Munich, Am Coulombwall 1, 85748 Garching, Germany*

[7] *Japan Synchrotron Radiation Research Institute, 1-1-1 Kouto, Sayo-cho, Sayo-gun, Hyogo 679-5198, Japan*

[8] *RIKEN SPring-8 Center, 1-1-1 Kouto, Sayo-cho, Sayo-gun, Hyogo 679-5148, Japan*

[9] *Center for Free-Electron Laser Science, Deutsches Elektronen-Synchrotron DESY, Notkestraße 85, 22607 Hamburg, Germany*

[10] *present address: Deutsches Elektronen-Synchrotron DESY, Notkestraße 85, 22607 Hamburg, Germany*

[11] *present address: Center for Synchrotron Radiation, Technical University of Dortmund, Maria-Goeppert-Mayer-Str. 2, 44227 Dortmund, Germany*

[12] *present address: European XFEL GmbH, Holzkoppel 4, 22869 Schenefeld, Germany*

[*] corresponding authors: michael.diez@xfel.eu, sebastian.schulz@desy.de, christian.bressler@xfel.eu

Characterization of the free-flowing liquid jet: Thickness

The measured interferometric signal does not only contain information on the relative arrival time of the X-ray and the optical laser pulse, as also the thickness of the sample can be extracted in-situ for every single X-ray shot based on the sine contribution of Eq. 1 in the Methods section of the main text. Its fitting parameters are the sine wave amplitude A, the sine wave frequency ν and its phase φ. The thickness of the liquid sheet is then extracted by the analysis of the observed interference pattern near the sharp edges: by counting the clearly visible fringes near the interferometric edge positions (usually the first ±2), it can be calculated as

$$d = (m_2 - m_1)\, \lambda_1\, \lambda_2 \,/\, (2\, (n_2\, \lambda_2 - n_1\, \lambda_1)) \qquad \text{(SE1)}$$

where $(m_2 - m_1)$ are the number of fringes counted (e.g., the interference maxima) at the corresponding wavelengths λ_i using their associated refractive indices $n(\lambda_i) = n_i$ with $i = 1, 2$.

In addition to the determination of the sheet's thickness in-situ as described above for every single X-ray shot, its thickness (and flatness) was also determined with a commercial device prior to the time arrival measurements. It is based on confocal achromatic imaging, where the light of a broadband ("white") source (typically an LED) is imaged through a chromatic lens yielding a dispersion of monochromatic light along the z-axis, i.e., the foci of different wavelengths are dispersed along the z-axis. Placing a thin transparent optic in this longitudinally dispersive region, being here the thin flat sheet jet, only two single wavelengths will be efficiently reflected into the chromatic lens, one from the front and the other from the backside of the sample. These two wavelengths are then imaged through a filtering pinhole, which suppresses all other wavelengths not in focus on the sample. A spectrometer determines both back-reflected wavelengths and the thickness of the sample is then calculated by relationship between the chromatic focusing distances and the recorded wavelengths. We used a commercial device for this purpose (Polytec/STIL TopSens CCS Prima with optical pen CL0-MG140), which allows to measure thicknesses in the range from sub-5 µm up to 100 µm at a working distance of approximately 2.7 mm. Because of the size of the device, this method cannot be used as an online thickness monitor and is used only prior to the actual arrival time measurements to set the desired sheet thickness.

Figure S1 a) shows the retrieved jet thickness for each recorded chirped pulse over a time span of 160 seconds, yielding (14 ± 2.4) µm. This is in close agreement with the (10 ± 1.1) µm extracted from the commercial thickness sensor (Fig. S1 b), although being measured independently and under slightly different conditions: the latter measurement had been carried out during the experiment's setup phase approximately 24 hours before the timing tool studies commenced. Its smaller mean value indicates changed conditions overnight, which also underlines the need to have as much in-situ information as possible during such an experiment and yields an average value

over a much longer measurement period of about 100 ms. Since the interference pattern used for the thickness measurement is taken exactly during the pump-probe shot and at the very same lateral position as the time-resolved laser-pump/X-ray probe measurement, the data in Fig. S1 a) represents the most accurate values for the real liquid jet thickness during such a pump-probe study, and this information can be useful in additional *a posteriori* correction of the longitudinal dimension of the interacting sample volume.

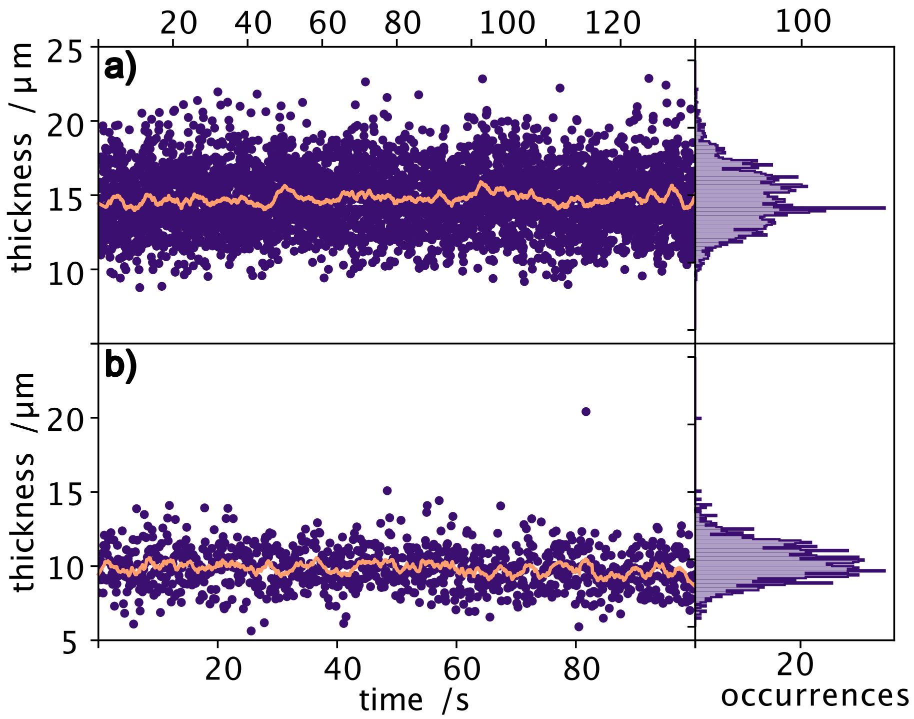

Supplementary Figure S1. Thickness of the flat sheet of the liquid jet, extracted from the measured spectral interference pattern alongside the actual timing data in the sample a) and measured prior to the experiment with a commercial confocal imaging device b). In both panels the orange line is indicating a moving average of the data as a guide to the eye.

Flow speed

The flow speed of the liquid jet is an important figure of merit for the MHz intra-train repetition rate of European XFEL (Ref. 5 of the main text) and for the upcoming LCLS-II (Ref. 34 of the main text). Ideally, the irradiated volume by the X-ray and the optical laser pulses are completely replaced for every shot. For instance, for a maximum size of 25 µm for both beams on the jet a linear flow speed of 25 m/s is required to accommodate a repetition rate of 1 MHz, corresponding to an X-ray pulse spacing of 1 µs in case of LCLS-II and similar for the 1.1 MHz special pulse pattern offered at European XFEL. For 4.5 MHz, the maximum intra-burst repetition rate at

European XFEL, corresponding to a minimum X-ray pulse spacing of 222 ns, jet flow speeds beyond 113 m/s are necessary. To determine the flow speed of our liquid jet, we treated the flowing sheet with an intense ultrashort laser pulse and then tracked the evolution of this distortion in time. For this investigation, the laser pulses were striking the liquid sheet at kHz repetition rates, and evolution of each impact was tracked with both fast recording and nanosecond gated cameras using time-lapse technique. In a first series of measurements, the back-illuminated liquid jet is imaged using a microscope objective onto the sensor of a high-speed camera (Photron FastCam SA4), which is capable of recording up to 500,000 frames per second (fps) with a minimum shutter opening time of 1 µs. This records the development of one single impact over a time period up to 110 µs. In a second series of measurements aiming to record the faster nanosecond time scales, we used a gated image intensifier (Hamamatsu C9538-03). It records triggered images at fixed time delays thus averaging over several individual shots for each time point. We reconstruct a time-lapse movie using different time delays over a range of 50 µs. Both techniques delivered a maximum flow speed of our liquid jet system (under stable conditions in terms of thickness and flatness) of up to approximately 60 m/s. While this already approaches the required refreshment rate at 4 MHz, it already fulfils the requirements for experiments at LCLS-II (1 MHz) and at European XFEL (for 0.5 MHz, 1.1 MHz and 2.25 MHz). Additional efforts are thus within reach to utilise fully refreshed samples at European XFEL's highest intra-burst repetition rate of 4.5 MHz.
